# Rapid Categorization of Human and Ape Faces in 9-Month-Old Infants Revealed by Fast Periodic Visual Stimulation

**DOI:** 10.1038/s41598-017-12760-2

**Published:** 2017-10-02

**Authors:** Stefanie Peykarjou, Stefanie Hoehl, Sabina Pauen, Bruno Rossion

**Affiliations:** 1 Department of Psychology, Heidelberg University, Heidelberg, Germany; 2 Face Categorization Lab, UC Louvain, Louvain-la-Neuve, Belgium; 3 Max Planck Institute for Human Cognitive and Brain Sciences, Leipzig, Germany

## Abstract

*This* study investigates categorization of human and ape faces in 9-month-olds using a Fast Periodic Visual Stimulation (FPVS) paradigm while measuring EEG. *C*ategorization response*s are* elicited only if infants discriminate between different categories and generalize across exemplars within each category. In study 1*, human or ape faces were presented as standard and deviant stimuli in upright and inverted trials*. Upright ape faces presented among humans elicited strong categorization responses, whereas responses for upright human faces and for inverted ape faces were smaller. *Deviant* inverted human faces did not elicit categorization. Data were best explained by a model with main effects of species and orientation. However, variance of low-level image characteristics was higher for the ape than the human category. Variance was matched to replicate this finding in an independent sample (study 2). Both human and ape faces elicited categorization in upright and inverted conditions, but upright ape faces elicited the strongest responses. *Again*, data were best explained by a model of two main effects. These experiments demonstrate that 9-month-olds rapidly categorize faces, and unfamiliar faces *presented among human faces* elicit increased categorization responses. *This likely reflects habituation for the familiar standard category, and stronger release for the unfamiliar category deviants*.

## Introduction

One of the most important visual challenges faced by young infants is to detect other human beings in their environment. Infants are surrounded by other humans most of the time, and are attracted by human faces in particular: For about 25% of their awake time, infants gaze at human faces^1^. Human faces form a homogeneous group of stimuli consisting of an oval shape with two eyes above a nose and a mouth. Given the high amount of exposure to faces and the homogeneity of exemplars of this category, it is not surprising that infants develop a categorical representation of faces from an early age^2^. However, the degree of specificity of this representation, in particular whether it differs for human and similarly looking nonhuman primate faces, remains unknown. The current study investigates this issue by testing visual categorization of human and ape faces in 9-month-old infants.

Perceptual categorization of human faces has been documented with brain and behavioural measures in adults. Human faces activate specialized regions along the ventral visual pathway with a right hemispheric advantage^3–5^, and elicit a right-lateralized face-sensitive event-related-potential (ERP) response peaking at ~170 ms, the N170^6^. It is increased in amplitude and latency for inverted faces^7–9^. Human individual face recognition is characteristically impaired for faces belonging to unfamiliar face categories, such as other species^10,11^ or other human groups, the “other-race” face effect^12^ for review^13^.

Several studies have compared the N170 in response to human and ape faces^14–16^. Carmel and Bentin^14^ observed shorter N170 peak latencies for human than ape faces. A similar effect was obtained by Itier and collegues^16^, who also observed that the inversion effect was more pronounced for human faces in latency and absent for ape faces in amplitude. Another study found smaller amplitude for human than monkey faces, and an inversion effect that was restricted to human faces^15^. The characteristics of the N170 for faces of different species have thus not been consistent across studies. Moreover, the N170 component is not present in infants, but two ERP components are considered as its precursors, the N290 and P400^17,18^. These components differ from the N170 in timing, scalp distribution, polarity (in case of the P400), and partly in response properties. This makes it difficult to predict how the species of faces may be reflected in infants’ electrophysiological responses compared to adults’.

Processing of human and ape faces has been compared repeatedly during the first year of life. Newborns do not show a preference for human or ape faces, but a preference for upright faces irrespective of species^19^. Whereas young infants discriminate individual ape faces similarly to human faces, from 6 to 9 months of age individuation of ape faces declines^20,21^ for similar results obtained with sheep faces, see^22^. Experience in individuating ape faces helps infants to maintain their ability to discriminate them at 9 months^21^. When older infants are given more time to process the faces, discrimination of unfamiliar face categories is still possible^23^.

Evidence for common categorization of human and ape faces (*i.e., jointly forming the category of* primate faces), as well as distinct categorization (i.e., human *vs*. ape faces *as different sub-categories of primate faces*) has recently been obtained in 9-month-old infants^24^. In this study, broad categorical repetition effects (face/non-face) were observed on the level of the early visual P1 component, *which was elicited with increased amplitude and decreased latency for all faces following house fronts compared to faces*. *In addition, a* species-specific repetition effect was observed on the level of the N290: *N290 amplitude and latency were enhanced for human targets following ape face adaptors, whereas amplitude and latency were decreased for ape targets following human face adaptors. This was taken to indicate that the N290 reflects activation of basic-level representations*. In another line of research, the two potentially face-sensitive infant ERP components, N290 and P400, were compared for human and monkey faces^15,25^. In all age groups tested (3-, 6-, and 12-month-olds), processing differences between the two face categories were observed, but they were not consistent across age-groups. A human face-specific increase in N290 amplitude for inverted faces has been obtained only in 12-month-olds^25^.

Several challenges make it difficult to draw conclusions from infant ERP studies *measuring average responses to human and ape faces* ^15,25^. First, these studies suffered from relatively high drop-out rates of 63–81%, which raises the question whether their results can be generalized. Second, human and monkey faces were presented in a between-subjects design so that every infant viewed only faces from one species. Therefore, infants were not required to categorize faces at all. Third, processing differences between the different face species were observed at every age tested. One may wonder whether such differences truly reflect perceptual categorization. For instance, it has been suggested that the human face-specific inversion effect on N290 amplitude in 12-month-olds reveals expert face processing^17^, but the inversion effect is no indication for categorical perception. To clearly demonstrate perceptual categorization, a paradigm is required that tests both discrimination between exemplars belonging to different categories, and generalization across exemplars belonging to the same category.

In addition, *expert perceptual categorization requires fast and automatic processing* ^26^, for,review. *In adults, categorization* is very rapid: Broad categorization as animal/no animal takes place within 150 ms^27,28^, at about the same time as the onset of the N170. This ERP component reliably differentiates faces and various animal and object categories^6,29^, forareview, see^30^. Concrete e.g., “face”, “car”, “dog”^31,32^; and abstract e.g., “living”, “non-living”^33,34^; categorization can even take place after having viewed an image for less than 50 ms. Moreover, face perception seems to be mandatory, that is, faces cannot be ignored even if it is required by the task^35–37^, and face subcategory (e.g., gender) judgements are not impaired by reduced attention^38^. Thus, it seems that face categorization occurs effortlessly in adults.

Recently, categorization in this sense (a rapid, automatic response including both discrimination and generalization) of human faces from many non-face visual objects has been demonstrated in adults with Fast Periodic Visual Stimulation (FPVS)^39^ while measuring electroencephalography (EEG). In this paradigm, highly heterogeneous images of human faces were periodically presented between diverse images of different biological and non-biological objects including animals. In 4–6-monht-old infants, human faces elicited a strong right-lateralized occipito-temporal categorization response^2^. *Similar to adults*, this response was driven by high-level representations, as it was not found for phase-scrambled images.

To evaluate whether infants have developed perceptual categories for human and ape faces, and to overcome limitations of previous ERP studies, we used a similar FPVS paradigm in the present study. FPVS has several advantages compared to standard ERP measures: (1) FPVS has a high signal-to-noise ratio, requiring short looking times so that only few trials are needed and few participants need to be excluded; (2) the different stimulus categories are embedded within one sequence and a categorization response will only be elicited if all (or most) exemplars are categorized, (3) and the categorization response can be defined and quantified objectively.

Here, we tested 9-month-old infants with sequences of human or ape faces as standard stimuli in which the respective other category was presented periodically as every 5^th^ image. At 9 months, behavioural work has demonstrated that individuation of ape faces has declined^20^ and ERP work has indicated that the two categories are discriminated when stimuli *are* presented in an upright position^24^. Accordingly, we predicted that 9-month-olds show a categorization response when presented with upright human versus ape faces. Whether categorization is similar for the two categories is an open question: *On the one hand, both human and ape deviant conditions require categorization of human and ape faces, making it likely that infants will show similar responses to the two conditions. On the other hand*, extensive experience with processing of human faces might support the process of activating an already existing categorical representation*, thereby increasing novelty responses to ape face deviants*.

Moreover, this study explored the contribution of low-level image characteristics to face species categorization. If these cues were fully sufficient to discriminate both face categories, we would expect similar categorization performance in upright and inverted conditions because low-level cues are identical in both cases. However, if categorization were based on higher-level visual representations and previous real-world experience, infants should show a stronger categorization response when looking at stimuli presented upright than at faces presented in an inverted orientation.

The FPVS paradigm allows us to determine categorization performance not only at the group level but also at the level of individual infants. Study 1 provides an initial investigation of rapid processing of upright and inverted human and ape faces at 9 months of age. Based on this pilot study we then optimize the stimulus set and specify hypotheses to test with an independent sample in study 2. Findings of both studies provide the basis for our conclusions.

## Study 1

### Material and Methods

#### Participants

Twenty-two 9-month-old infants were tested (10 female, mean age = 9 months, 12 days, SD = 9 days). *In accordance with the terms provided by the local ethics committee of Heidelberg University*, verbal informed consent *was obtained* from their caretaker*s*. Two additional infants were tested but excluded (one due to excessive crying, one due to insufficient data quality). All methods were carried out in accordance with relevant guidelines and regulations. The general procedure has been approved by the local ethics committee of Heidelberg University.

#### Stimuli/Presentation

Infants were presented with sequences of human and ape faces. Images were displayed in upright and inverted orientations in subsequent trials. The presentation was similar to recent studies employing the FPVS technique^2,40,41^. Fifteen images each of human and ape faces were presented. Human face images were taken from standard face databases^42,43^, *whereas ape face images were collected through google search. All faces showed a neutral emotional expression, were presented in full-frontal view, and cropped to an oval shape excluding outer facial contours and, in the case of human faces, hair. Cropping was performed in order to increase infants’ focus on inner facial features and to ensure that categorization was not based on facial contours varying between species (e.g., the transition from smooth skin to hair in humans versus the continuous presence of facial hair in apes)*. Mean luminance was equalized across categories.

**Figure 1:**
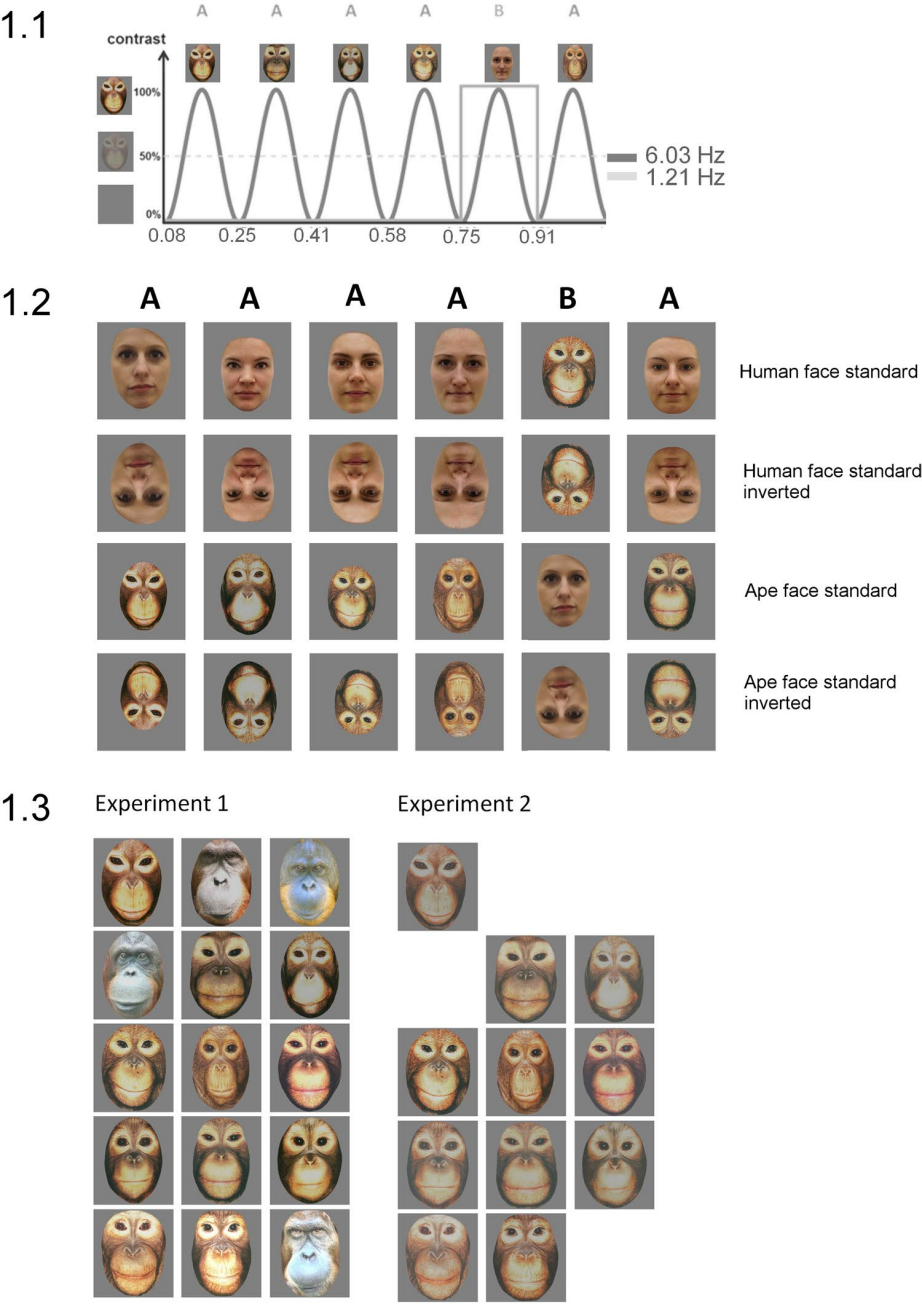
Schematic illustration of the experimental paradigm, conditions and stimuli. 1.1 Experimental paradigm. Images were presented by sinusoidal contrast modulation at a rate of 6.03 cycles per second = 6.03 Hz (1 cycle ≈ 170 ms). Ape or human faces stimuli were presented at every 5^th^ cycle (**B**) in subsequent trials (6.03/5 Hz = 1.21 Hz). The respective other category was presented as standard stimulus. Human faces images were not for publication and were thus replaced for all figures. 1.2 Conditions. The standard category (ape face, human face) was changed between-subjects. Note also that the stimuli changed size (range +/− 10%) at every stimulation cycle. The orientation of images (upright, inverted) was manipulated within-subjects. 1.3 Stimuli. Whole sets of ape face images used in the two experiments.

Images were displayed on a light grey background. Infants sat at a looking distance of 60 cm, and pixel size was 550 (width) × 607 (height), corresponding to approximately 12 × 15 degrees of visual angle. Images changed size (+/−10%) at every stimulation cycle. MATLAB 7.8 (The Mathworks) with PsychToolbox (http://psychtoolbox.org/) was used for stimulus display. Stimulus sequences were presented at a fixed rate of 6.03 cycles per second (*F* = 6.03 Hz; base stimulation frequency) through sinusoidal contrast modulation^44^. Each cycle lasted 166 ms (i.e., 1000 ms/6.033). Trials started with a uniform grey background from which an image appeared as contrast increased. The stimulation was gradually faded in by progressively increasing the modulation depth from 0% to 100% maximum contrast level (and faded out vice versa). Each stimulus reached full contrast at 83 ms, then contrast was decreased at the same rate. At fixed intervals of every 5th image, a stimulus from the other category was introduced, creating a trial sequence containing category changes at a frequency of 1.21 Hz (6.03 Hz/5; i.e., A = Ape; H = Human: HHHHAHHHHA..…). EEG amplitude at this frequency (*F*/5 = 1.21 Hz) and its harmonics (i.e., 2 *F*/5 = 2.41 Hz, 3 *F*/5 = 3.62 Hz…) was used as an index of the visual system’s categorization of face species^45^. The schematic stimulation course is illustrated in Fig. 1.

Four types of trials were presented: ape face deviant (with human face standard), human face deviant (with ape face standard), and likewise versions of these trials with pictures inverted. For half the sample, human faces served as standard, for half the sample it was vice versa. Stimulus orientation was varied within-subject across trials (four consecutive trials upright, then four trials inverted, four upright, four inverted). *As we were primarily interested in processing of upright faces, corresponding trials were always presented first to increase the number of trials available*. Stimulus order was randomized for each trial with the exception that no stimulus could be repeated immediately. Between trials, short breaks were provided if needed. Overall, testing took about 10 minutes.

#### Procedure

Infants were seated at a looking distance of approx. 60 cm from the computer screen on their caregiver’s lap. Each trial consisted of a blank screen (random, min. 5 seconds), a 2-second fade-in, a stimulation sequence for 20 seconds, and a fade-out of 2 seconds. Stimulus fade-in and fade-out were introduced to avoid surprise reactions, abrupt eye-movements or blinks.

Triggers were sent via parallel port at the start of the each sequence and at the minimum of each cycle (grey background, 0% contrast). Trigger accuracy was registered by a photodiode located in the upper left corner of the monitor. During the entire stimulation, looking-behavior was video-taped and coded offline. Trials were initiated manually when participants looked attentively at the screen and showed an artifact-free EEG signal.

#### EEG Recordings and Analyses

EEG measures were obtained applying a BrainProducts actiCap (Gilching, Germany) with 32 active Ag-AgCl electrodes arranged according to the 10-10-system and a right mastoid reference. Sampling rate was set at 250 Hz and the EEG signal was amplified via a BrainAmp amplifier. Impedances were considered acceptable if < 20 kΩ. Recordings were acquired in a dimly-lit and quiet room.

EEG Preprocessing. All EEG processing steps were carried out using Letswave (http://nocions.webnode.com/letswave) and Matlab 2012b (The Mathworks) and followed the procedure described in several recent studies^40,46^. EEG data was first band-pass filtered at 0.1–100 Hz using a Butterworth filter with a slope of 24 dB/octet. Filtered data was then segmented 2 seconds before and after the sequence, resulting in 28-second segments (−2 s–26 s). Next, noisy channels were identified and pooled from surrounding channels (for a maximum of 2 channels) and a common average reference computation was applied to all channels.

Frequency domain analysis. Preprocessed data segments were cropped to an integer number of 6.03 Hz cycles beginning 2 seconds after onset of the trial until approximately 20 seconds, just before the stimulus fade-out (120 cycles, 4973 time bins in total ≈ 19.892 s). The first two seconds of each trial were excluded to avoid any contamination by the initial transient responses. For each condition, trials were averaged in the time-domain for every individual participant. Averaging was performed to increase the signal-to-noise ratio (SNR) by reducing EEG activities non-phase-locked to the stimulus. Then a Fast Fourier Transform (FFT) was applied to these averaged segments to extract amplitude spectra for all channels (square root of sum of squares of the real and imaginary parts divided by the number of data points). Frequency analysis yielded spectra with a high frequency resolution of 0.0503 Hz (1/19.892 s).

To measure the magnitude of activity at pre-defined bins of interest, baseline corrected amplitudes were computed by subtracting the average amplitude of 12 surrounding bins (6 on each side, excluding the immediately adjacent bins) from every frequency bin^40,46^. *For the base rate response, only occipital channels (O1, O2, Oz) were considered, for the categorization response, all occipito-temporal channels (P7, P8, PO9, PO10, O1, O2, Oz) were considered*. Z-scores were calculated as the difference between amplitude at the frequency of interest and mean amplitude of 12 surrounding bins divided by the standard deviation of the 12 surrounding bins^41^. Threshold of significance was placed at Z-score 1.64 (*p* < 0.05, one-tailed). SNRs were computed by dividing the signal by the amplitude at the 12 neighboring frequency bins. Note that in the current study, 12 rather than 20 bins as in previous studies^40,41^ were used to estimate noise variance. Due to shorter recording time in infants compared to adults (26 versus 66 second trials), the frequency resolution in this study is lower than in previous reports. In order to avoid including low parts of the spectrum that are inherently contaminated by higher levels of biological noise, the number of bins for noise variance estimation was reduced.

Only trials with a significant response at the base frequency (6.03 and/or its harmonic 12.07) were used. On average, participants viewed 10 trials (*M* = 10.41; *SD* = 2.8), of which one trial (*M* = 1.36; *SD* = 1.7) was excluded due to a non-significant base rate response. There was no difference in the number of trials in the human (*M* = 10.4; *SD* = 3.4) and ape conditions (*M* = 10.4; *SD* = 2.1; *p* > 0.05), but participants saw more upright (*M* = 6.2; *SD* = 1.8) than inverted trials (*M* = 4.2; *SD* = 1.2; *p* < 0.001). To ensure that results could not be explained by differences in trial numbers, additional analyses were performed using a matched number of upright and inverted trials (trials from the upright condition randomly excluded). The results pattern conformed to the analyses on all trials. Additionally, trials were selected based on looking time, which was coded offline from the video. 20% of trials were double-coded, with an intraclass correlation (ICC) coefficient of 0.98. When using only trials in which looking time was > 50%, the results pattern was similar to the main analyses.

Statistical analyses were performed using baseline corrected amplitudes (summed up to the highest consecutively significant harmonic^46^). For the categorization response, 1.21 Hz and harmonics were summed up to the 11^th^ harmonic, but excluding the 5^th^ and 10^th^ harmonics which correspond to the base frequency. For the base stimulation response, 6.03 Hz and harmonics were summed up to the 6^th^ harmonic. Channels of interest were defined based on scalp topographies: P7, P8, PO9, PO10, O1, O2, Oz for the categorization response and O1, O2, Oz for the base response.

Z-scores were *calculated* to determine whether a significant response was obtained in each condition *after summing across harmonics*. Conditions were compared using baseline corrected amplitudes in a JZS Bayes factor repeated measurement analysis of variance (rmANOVA) with default prior scales^47,48^. Factors were species (2: human deviant, ape deviant) * orientation (2: upright, inverted). Preliminary analyses indicated that there was no main effect or interaction with electrode, so an average of all seven electrodes (categorization response) or three electrodes (base response) was calculated and used in the statistical analyses. The Bayes factor rmANOVA provides a more conservative test than the standard rmANOVA and estimates probability for models based on the null and alternative hypotheses.

We hypothesized that upright images would elicit stronger categorization responses than inverted images. *We did not have strong predictions regarding categorization differences between human and ape deviants, as both conditions require categorization of human and ape faces. However, extensive experience with human faces may enhance adaptation for human standards, which would lead to stronger categorization responses for ape deviants*.

### Results

#### Categorization Response

**Figure 2:**
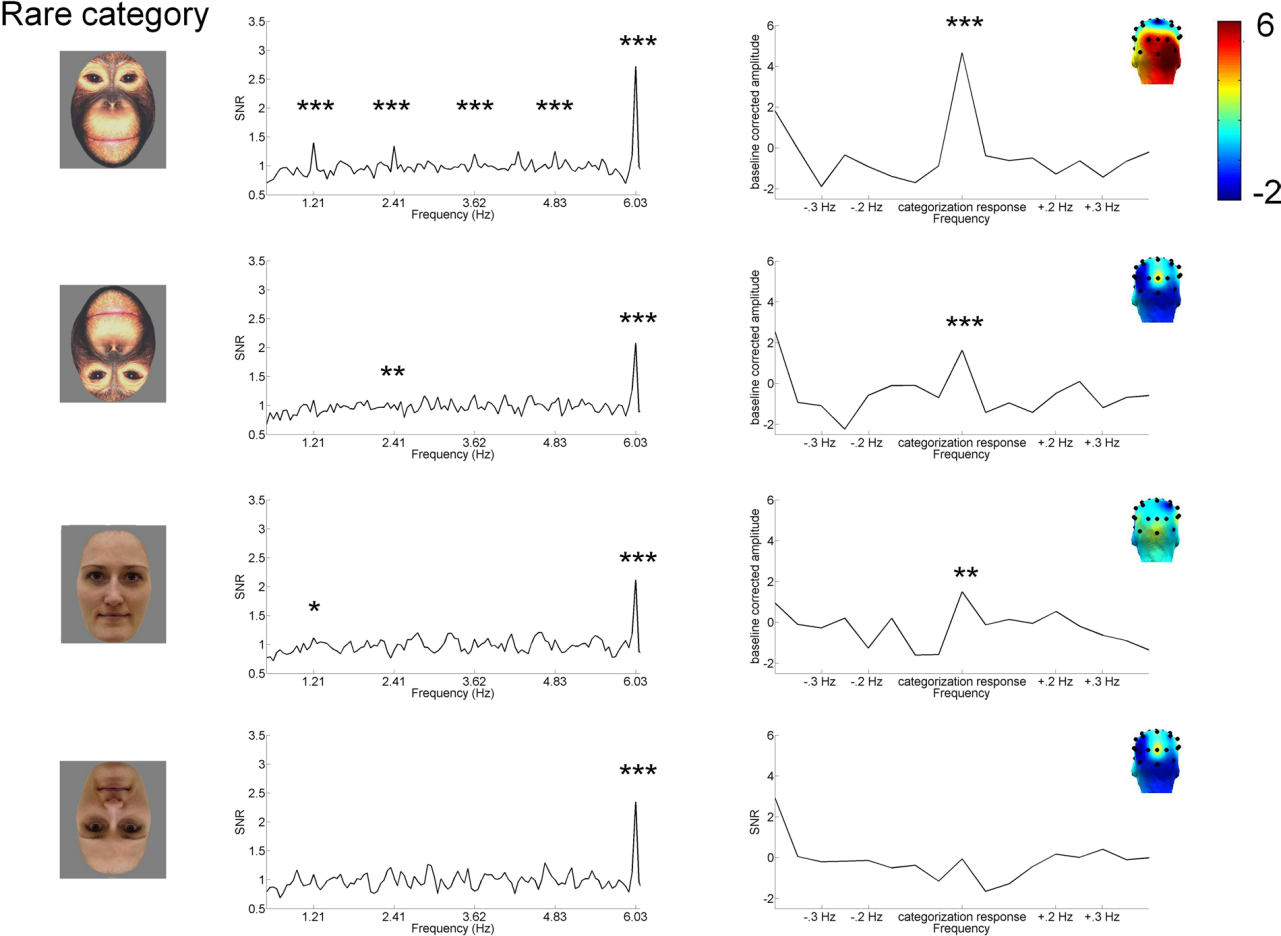
Results of experiment 1. SNR of categorization response (1.21 Hz, 2.41 Hz, 3.62 Hz, 4.83 Hz) and of base response (6.03 Hz) and summed baseline corrected amplitude of categorization response (harmonics 1–11, excluding base response at 5^th^ and 10^th^ harmonic). Data has been averaged across electrodes (P7, P8, PO9, PO10, O1, O2, Oz) and grand-averaged across participants. There was no difference between conditions in the base response. The categorization response was observed for rarely presented upright ape faces, inverted ape faces, and upright human faces, but was strongest for upright ape deviants. *p < 0.05, **p < 0.01, ***p < 0.001.

The categorization response (response at 1.21 Hz and harmonics) was observable in the grand-averaged data when upright ape faces were presented as deviant stimuli among human faces (SNR 1.37, Z > 3.11, *p* < 0.01; see Fig. 2 and Table 1). It was spread over occipital channels, with a slight right-hemispheric advantage. When looking at single infants, a significant response was obtained in six out of 11 infants in that condition (*Z*s > 3.11, *p*s < 0.001). There also was a categorization response for upright human deviant faces (SNR 1.08, Z > 2.33, *p* < 0.05) and inverted ape deviants (SNR 1.20, Z > 3.11, *p* < 0.01). In analyses of individual responses, a categorization response was observed for inverted ape among human faces in six of 11 infants (*Z*s > 2.33, *p*s < 0.01), and for upright human among ape faces in seven of 11 infants (*Zs* > 1.64, *p* < 0.05). No categorization response was observed for inverted human deviant faces on grand-averaged data (*p* > 0.05), but one infant among 11 showed a categorization response for inverted human faces among ape faces (*Z* > 1.64, *p* < 0.05).

**Table 1:** Baseline corrected amplitude (bca) means and standard deviations (SD), Z-score and signal-to-noise ratio (SNR) ranges for individual categorization and base rate responses in experiment 1.

Response	deviant category	orientation	bca mean	bca SD	Z-score range	SNR range	N
categorization (1.21 + harmonics)	Ape	upright	4.67	4.28	0.21–8.74	1.01–2.80	11
		inverted	1.63	4.13	−1.59–6.63	0.80–1.81	11
	human	upright	1.49	3.60	−1.71–8.57	0.83–1.73	11
		inverted	−0.06	2.02	−1.76–2.01	0.80–1.35	11
base (6.03 + harmonics)	Ape	upright	2.79	2.22	0.21–36.45	1.05–8.91	11
		inverted	2.46	2.67	−0.11–26.76	0.99–7.56	11
	human	upright	2.68	1.24	1.81–17.64	1.31–5.00	11
		inverted	3.11	1.77	1.08–21.95	1.18–6.04	11

The categorization response was summed across harmonics 1–11 (excluding harmonics 5 + 10), the base response across harmonics 1–6.

The Bayes rmANOVA revealed that the model with a main effect of orientation was preferred to the null model by a Bayes factor of 2.31. This provides marginal evidence^49^, Appendix B for the hypothesis that categorization responses were stronger for upright images irrespective of species (upright *M* = 3.08 µV; *SD* = 4.2; inverted *M* = 0.78 µV; *SD* = 3.3). Moreover, the model with two main effects (species and orientation) was preferred to the null model by a Bayes factor of 3.07, providing moderate evidence that categorization responses differed between upright and inverted conditions and between human and ape deviants (ape face deviants *M* = 3.15 µV; *SD* = 4.4; human face deviants *M* = 0.72 µV; *SD* = 3.0). The difference between the model with a main effect of orientation and the one with main effects of species and orientation was only marginal (Bayes JZS = 0.75) but, went in favor of the model with two main effects. The model with two main factors was also marginally preferred over a model with the main factor species (Bayes JZS = 2.27) and over a model with two main factors and an interaction term (Bayes JZS = 2.19).

#### Base Response

A strong response to the base visual stimulation was observed in all conditions (all SNRs > 2.1, all Zs > 10, see Table 2). It was centered on channel Oz and spread over O1 and O2. This response was significant in nine of 11 infants for upright ape faces (*Z*s > 3.11 *p*s < 0.001), in eight of 11 infants for inverted ape faces (*Z*s > 3.11, *p*s < 0.001), in all 11 infants for upright human faces (*Z*s > 1.64, *p*s < 0.05), and in nine of 11 infants for inverted human faces (*Z*s > 2.33, *p*s < 0.01).

**Table 2:** Baseline corrected amplitude (bca) means and standard deviations (SD), Z-score and signal-to-noise ratio (SNR) ranges for individual categorization and base rate responses in experiment 2.

Response	deviant category	Orientation	bca mean	bca SD	Z-score range	SNR range	N
categorization (1.21 + harmonics)	ape	Upright	5.97	3.19	1.39–26.84	1.18–3.13	10
		Inverted	2.73	2.94	−1.04–18.84	0.91–2.23	10
	human	Upright	3.23	2.24	0.05–4.75	1.00–1.52	9
		Inverted	0.07	1.83	−1.22–1.66	0.87–1.26	9
base (6.03 + harmonics)	ape	Upright	4.40	3.05	1.21–25.06	1.27–7.63	10
		Inverted	3.86	3.55	1.32–22.01	1.29–8.03	10
	human	Upright	2.18	1.79	−0.99–20.67	0.87–4.72	9
		Inverted	1.54	1.31	0.24–4.88	1.05–2.22	9

The categorization response was summed across harmonics 1–14 (excluding harmonics 5 + 10), the base response across harmonics 1–4.

The Bayes rmANOVA confirmed that there were no differences between conditions (JZS Bayes factors < 1 > 0.3).

### Discussion

In study 1, we explored 9-month-old infants’ rapid categorization of human and ape faces. As a group, infants showed a strong categorization response for upright ape faces presented among human faces, which was spread over the occipital cortex. Moreover, this response *reached significant threshold* in individual averages of six out of 11 infants. Categorization was also observed for upright human face deviants and inverted ape face deviants. Categorization responses best fit a model with main factors of species and orientation, indicating that categorization of ape faces and upright images was stronger than of human faces and inverted images. Thus, this study reveals that 9-month-old infants’ face species categorization relies on high-level visual perception and goes beyond mere perception of low-level image characteristics.

Moreover, this initial exploration of infant face categorization revealed an asymmetry, with stronger categorization responses for deviant ape faces. Before we can turn to discussing high-level explanations for this finding, however, low-level confounds should be ruled out. The asymmetry cannot be explained by a general difference of attention in human and ape standard trials. This was verified using two measures: (1) The response to the base stimulation frequency (6.03 Hz) did not differ between human and ape standard trials. (2) Video-coding confirmed that infants looked equally long at human (*M* = 16.11, *SD* = 5) and ape (*M* = 15.49 s, *SD* = 3.7) standard trials (*p* > 0.6). Therefore, we have no indication for differential attention to trials with different standard categories.

Likewise, categorization of ape from human faces cannot be attributed to low-level image characteristics, as inverting faces reduced categorization overall. Interestingly though, the categorization asymmetry was observed in inverted trials as well. Moreover, regarding individual infants’ responses, six infants showed a *significant* categorization response for rarely presented inverted ape faces, whereas only one infant categorized rarely presented inverted human faces. This raises the question whether some low-level cues may have biased infants to categorize ape, but not human faces. Visual examination of our images indicated that the heterogeneity of ape faces was larger than that of human faces. Whereas human faces were taken from face databases, ape faces were collected from free images via google search, and were thus more likely to vary. We extracted luminance and size values and statistical analyses confirmed that the standard deviations of both measures were larger for ape than human faces, while there was no difference in mean luminance and size. The larger variability of ape faces may have contributed to the asymmetrical categorization observed here: It might have been more difficult for infants to form a category of ape faces from which human faces could be distinguished. In comparison, detecting ape faces among the more homogeneous group of human faces might have been easier.

Therefore, we edited the images and matched the heterogeneity of face categories to examine categorization of those controlled stimuli in study 2. We based our hypotheses on study 1 and thus expected best model fit for a model with two main factors, orientation and species, reflecting stronger categorization responses for ape face deviants and upright conditions. These a priori hypotheses were evaluated using a rmANOVA. Thus, study 2 provides a test whether similar categorization patterns as in study 1 will be observed in an independent sample with controlled images.

## Study 2

### Material and Methods

#### Participants

Nineteen 9-month-old infants were tested (11 female, mean age = 9 months, 16 days, SD = 8 days) after obtaining verbal informed consent from their caretaker. Six additional infants were tested but excluded (three due to excessive crying, two due to insufficient data quality, and one due to rhythmic noise).

#### Stimuli/Presentation

The presentation was identical to study 1. From the stimuli presented in study 1, four images of ape faces were excluded because they were physically very different from the other ape faces, leaving 11 ape images. The number of human face images was matched by randomly excluding four images. Images were edited so that luminance means and SDs as well as pixel size means and SDs were equalized between the two categories. Ten infants watched the presentation with human faces as standard, nine with ape faces as standard.

#### Procedure

Procedure was identical to study 1.

#### EEG Recordings and Analyses

EEG recordings and analyses were identical to study 1. On average, participants viewed 11 trials (Mean = 10.80, SD = 2.5), of which one trial (Mean = 1.32, SD = 1.2) was excluded due to a non-significant base rate frequency. There was no difference in the number of trials in the human standard (*M* = 11.4; *SD* = 2.8) and ape standard conditions (*M* = 10.1; *SD* = 2.2; *p* > 0.05), but participants watched more upright (*M* = 6.3; *SD* = 1.6) than inverted trials (*M* = 4.5; *SD* = 1.3; *p* < 0.001). Similar to experiment 1, trials from the upright condition were randomly excluded to match the number of upright and inverted trials. The results pattern from this additional analysis conformed to the analyses on all trials, while giving a stronger effect of orientation.

Comparisons between conditions were performed in the same manner as in study 1. Baseline corrected amplitudes were summed up to the highest consecutively significant harmonic. For the categorization response, 1.21 Hz and harmonics were summed up to the 14^th^ harmonic, but excluding the 5^th^ and 10^th^ harmonics which correspond to the base frequency. However, when analyzing an average of channels P7, P8, PO9, PO10, O1, O2, and Oz, harmonics 1 and 2 were not significant. In an additional analysis, these two harmonics were excluded and analyses were run using a sum of harmonics 3–14. The results pattern confirmed the one obtained with harmonics 1–14, while giving a stronger effect of orientation. For the base response, 6.03 Hz and harmonics were summed up to the 4^th^ harmonic. Channels of interest were defined based on scalp topographies and conformed to the channels employed in study 1: P7, P8, PO9, PO10, O1, O2, Oz for the categorization response and O1, O2, Oz for the base response.

Preliminary analyses indicated that there was no main effect or interaction with electrode, so an average of all seven electrodes (categorization response) or three electrodes (base response) was calculated and used in the statistical analyses. The hypothesis that categorization responses would be strongest for upright ape deviants was tested using a Bayes rmANOVA with species (2: human deviant, ape deviant) * orientation (2: upright, inverted) as factors.

## Results

### Categorization Response

**Figure 3:**
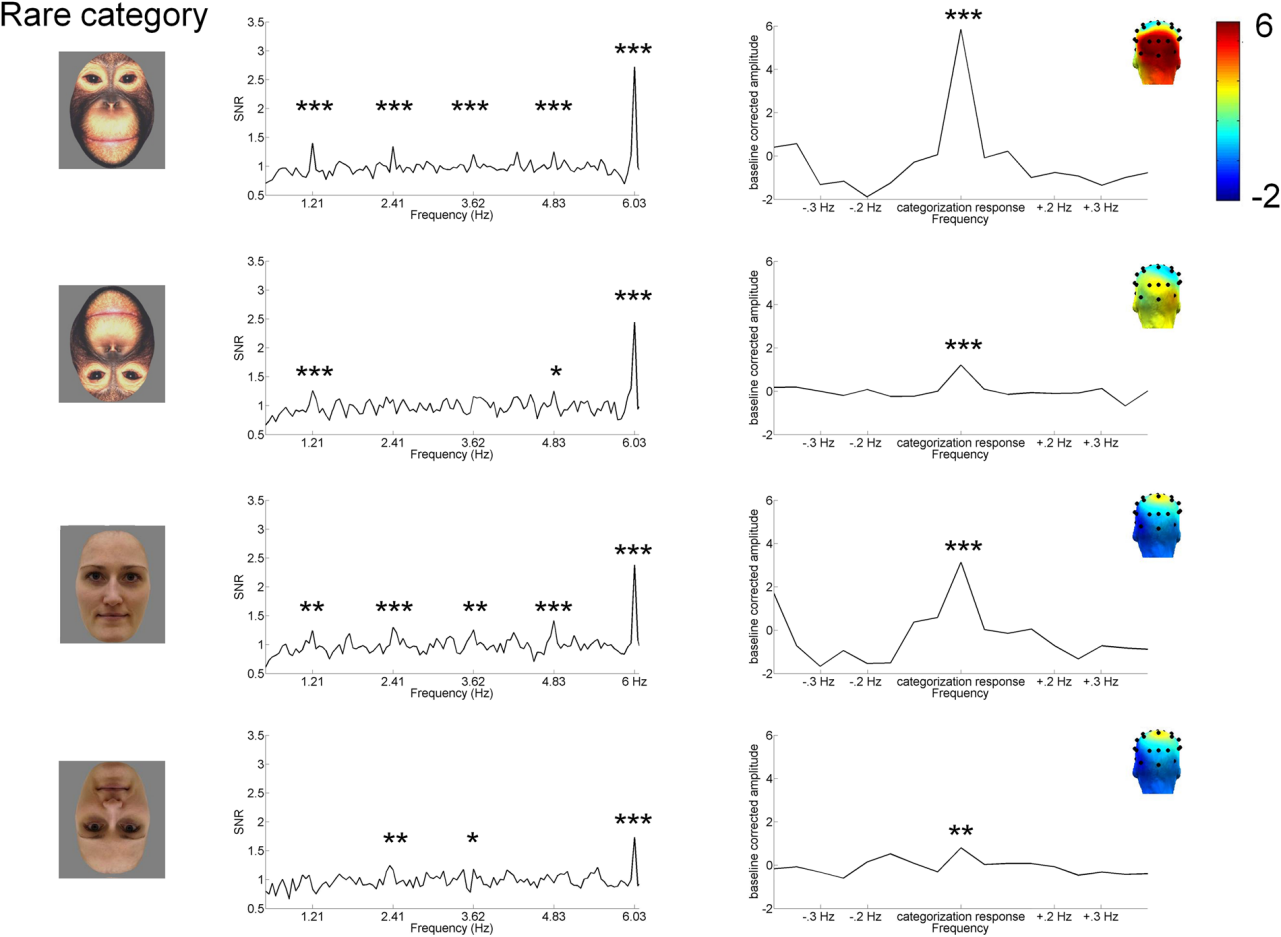
Results of experiment 2. SNR of categorization response (1.21 Hz, 2.41 Hz, 3.62 Hz, 4.83 Hz) and of base response (6.03 Hz) and summed baseline corrected amplitude of categorization response (harmonics 1–14, excluding base response at 5^th^ and 10^th^ harmonic). Data has been averaged across electrodes (P7, P8, PO9, PO10, O1, O2, Oz) and grand-averaged across participants. There was no difference between conditions in the base response. The categorization response was observed in all conditions, but was strongest for upright ape deviants. *p < 0.05, **p < 0.01, ***p < 0.001.

The categorization response (response at 1.21 Hz and harmonics) was observable in the grand-averaged data when upright ape faces were presented as deviant stimuli among human faces (SNR 1.59, Z > 3.11, *p* < 0.01; see Fig. 3 and Table 2) spread over the occipital cortex. Moreover, a significant response was obtained in nine out of 10 infants (*Z*s > 1.64, *p*s < 0.05). There also were categorization responses in the other three conditions (upright deviant human faces SNR = 1.26, Z > 3.11, *p* < 0.01; inverted deviant ape faces SNR = 1.21, Z > 3.11, *p < *0.01; inverted deviant human faces SNR = 1.10, Z > 2.33, *p* < 0.05). In analyses of individual responses, a categorization response was observed for upright human face deviants in seven of nine infants (Z*s* > 2.33, *ps* < 0.01), for inverted ape face deviants in five of 10 infants (Zs > 1.64, *p*s < 0.05), and for inverted human face deviants in one of nine infants (Z > 1.64, *p* < 0.05).

The Bayes rmANOVA revealed that the model with main effects of species and orientation was preferred to the null model by a Bayes factor of > 100. Compared to the model with a main effect of only orientation, it was preferred by a factor of 4.11, and compared to a model with only species by a factor of > 100. This provides conclusive evidence that the model of two main effects (species and orientation) fits the data better than the null model and the model with species only. Compared to the model with only a main effect of orientation, the two main factors model was moderately preferred. The model with two main factors was also conclusively preferred over a model with the main factor species (Bayes JZS > 100), and marginally over a model with two main factors and an interaction term (Bayes JZS = 2.42). Categorization responses were stronger for upright images irrespective of species (upright *M* = 4.67 µV; *SD* = 3.0; inverted *M* = 1.47 µV; *SD* = 2.8) and for ape face deviants than human face deviants irrespective of orientation (ape face deviants *M* = 4.35 µV; *SD* = 3.4; human face deviants *M* = 1.65 µV; *SD* = 2.6).

### Base Response

A strong response to the base stimulation was observed in all conditions (all SNRs > 1.65, all Zs > 1.96, except for human faces inverted where Z = 1.54, see Table 2). It was centered on channel Oz and spread over O1 and O2. This response was significant in nine of 10 infants for upright ape faces (*Z*s > 3.11, *p*s < 0.001), in nine of 10 infants for inverted ape faces (*Z*s > 1.64, *p*s < 0.05), in seven of nine infants for upright human faces (*Z*s > 1.64, *p*s < 0.05), and in eight of nine infants for inverted human faces (*Z*s > 2.33, *p*s < 0.01).

The Bayesian ANOVA confirmed that there were no differences between conditions (JZS Bayes factors < 1 > 0.3).

## Discussion

We tested 9-month-olds’ categorization of *highly controlled images of* human and ape faces using an FPVS paradigm and observed categorization responses that were similar to study 1. Ape faces presented among human faces elicited a strong categorization response over occipital areas, and human faces *presented among ape faces* elicited a smaller categorization response. Categorization of inverted images was much reduced, providing strong evidence that face species categorization in 9-month-old infants is not based on low-level cues.

Similar to study 1, we observed a categorization asymmetry where infants showed a stronger categorization response for rarely presented ape than human faces. Categorization in this study cannot be explained by low-level factors. The categorization response for inverted images was reduced, irrespective of face species. Moreover, the variance of luminance and size was matched in the two conditions, ruling out the possibility that increased variance of ape faces interfered with *detecting* human *among ape* faces, as might have been the case in study 1. The categorization asymmetry observed here cannot be explained by a general difference in attention for human and ape deviants, either, as there was no difference in the base rate response for respective trials. Alternative accounts for asymmetrical categorization will be considered in the General Discussion.

## General Discussion

Together, the two studies presented here provide evidence that 9-month-old infants can categorize upright faces according to species at a high speed, that is, in less than 170 ms, allowing only one fixation on each image. In study 1, we ran an initial investigation of human and ape face categorization and observed an *occipito-temporal* categorization response for upright face images. Categorization responses were stronger *when ape faces were presented as deviants among human faces than vice versa*. As the ape face image set had larger variability of luminance and size, it might have been more difficult to detect deviant human faces among the ape faces*; therefore*, we matched low-level stimulus characteristics and ran study 2. The data from this independent sample confirmed that infants’ rapidly categorized upright faces according to species. Again, *the categorization response* was stronger for deviant ape faces *presented among humans*.

We took great care to evaluate the contribution of low-level image characteristics to categorization. Infants looked equally long at trials with ape and human deviants (and at upright and inverted trials), so we have no indication that attention was increased in any condition. Moreover, the base rate response, a direct measure of neural activation in response to general visual stimulation, did not differ between conditions. Most importantly, we ran inverted versions of trials in which low-level characteristics are exactly the same as in upright trials. Whereas infants also categorized inverted faces (only ape deviants *among human standards* in study 1, ape and human deviants in study 2), this response was smaller than for upright faces. However, the categorization asymmetry was observed in upright and inverted conditions, so despite all controls, we cannot fully rule out the possibility that low-level factors inherently associated with the two face species *contributed to the categorization asymmetry*.

At 9 months of age, infants have acquired extensive experience with processing human faces^20,21^. Their experience with human faces may have allowed infants to categorize faces at a high speed in the current study, and may have enhanced categorization responses to the unfamiliar category of ape faces. Extensive experience with human faces may speed up the process of activating a formerly developed categorical representation, *while* exemplars that do not match this well defined representation (i.e., ape faces) elicit novelty responses. Such a novelty response would not be reflected in looking time or the base rate response, as those measures reflect processing during complete trials and cannot be compared for single stimuli. To further explore whether familiarity with one of the face categories is sufficient to elicit skewed categorization, it would be helpful to compare categorization of two unfamiliar face categories, for instance other-race faces and ape faces, at the end of the first year of life. *In general, future studies should address how categorization of the standard and the deviant category contribute to the strength of the categorization response*.

Asymmetrical categorization of human stimuli has been reported before in behavioral tasks^50,51^. In these studies, 3-4-month-old infants formed a category of humans (represented with head and body information) that included other animals, but formed a category of horses that excluded humans and other animals. This effect was restricted to conditions where head and body information was present, and not observed when only the head was presented^51^. No asymmetry was observed in an ERP paradigm on human-animal categorization^52^. Thus, though asymmetrical categorization of humans has been observed previously, these studies employed very different methods than the current study.

Future work should use a similar approach to test whether younger infants who have not yet developed a specialized face processing system for human faces (i.e., 6-month-olds^21^), show similar categorization of human and ape faces. *We would expect categories of human and ape faces to emerge gradually during development, with weaker categorization responses in younger infants. Expert processors (i.e., adults), in contrast, should show very clear categorization responses for human and ape faces. Moreover, we would expect all adults to show significant categorization of faces according to species, whereas it was significant here for only a subset of the 9-month-old infants (54–90% in the different upright conditions). Note that this does not mean that the response is completely absent for the other infants, but that it does not reach the significance threshold at the individual level (summed across harmonics, averaged across electrodes). However, their data contribute to the clear effects observed at the group level, which is the only effect considered in typical behavioral and electrophysiological studies in infants. Nevertheless, these differences could also reflect genuine developmental differences between individual infants at this age, who may develop categorization skills at slightly variable times. A longitudinal design would be best suited to investigate this hypothesis, ideally complemented with data on individual differences in behavioural categorization*.

Categorization in the current study occurred rapidly, that is, after seeing each image for only about 130 ms, with a stimulus onset asynchrony (SOA) of 170 ms. As a novel image faded in right after the previous one had faded out, stimulus processing was interrupted after 170 ms. Face species categorization was thus based on only gaze fixation by stimulus. Previous studies on human and ape face processing have employed presentation times of at least 500 ms^15^, and image presentation was followed by an ISI so that processing could continue. Overall, behavioural and ERP studies on categorization require much longer presentation times about 15 seconds in behavioral tasks, and between 500 and 1,500 ms in ERP tasks^17,50,53–55^. Thus, the categorization response observed in the current study demonstrates that high-level representations can be activated much faster than previously suspected in the infant’s brain, that is, within about 170 ms.

To sum up, the current study demonstrated rapid categorization of faces according to species in 9-month-old infants in two independent samples. Categorization was stronger for upright than inverted images, revealing that infant categorization is not based on low-level image characteristics but reflects high-level perception. While infants showed a strong categorization response *when ape faces were presented among humans*, a smaller response was observed for *human faces presented among apes*. It seems likely that *the greater familiarity of human faces induced stronger repetition effects for human standards, while enhancing detection of ape deviants*. Thus, extensive experience with human faces enables infants to categorize even unfamiliar face categories at a single glance.

## References

[1] Sugden NA, Mohamed‐Ali MI, Moulson MC (2014). I spy with my little eye: Typical, daily exposure to faces documented from a first‐person infant perspective. Developmental psychobiology.

[2] de Heering A, Rossion B (2015). Rapid categorization of natural face images in the infant right hemisphere. Elife.

[3] Haxby JV, Hoffman EA, Gobbini MI (2000). The distributed human neural system for face perception. Trends in Cognitive Sciences.

[4] Kanwisher N, McDermott J, Chun MM (1997). The fusiform face area: a module in human extrastriate cortex specialized for face perception. J Neurosci.

[5] Rossion B, Hanseeuw B, Dricot L (2012). Defining face perception areas in the human brain: A large-scale factorial fMRI face localizer analysis. Brain Cognition.

[6] Bentin S, Allison T, Puce A, Perez E, McCarthy G (1996). Electrophysiological studies of face perception in humans. J Cognitive Neurosci.

[7] Rossion B (1999). Spatio-temporal localization of the face inversion effect: an event-related potentials study. Biol Psychol.

[8] Eimer M (2000). Effects of face inversion on the structural encoding and recognition of faces. Evidence from event-related brain potentials. Cognitive Brain Res.

[9] Itier RJ, Taylor MJ (2002). Inversion and contrast polarity reversal affect both encoding and recognition processes of unfamiliar faces: a repetition study using ERPs. Neuroimage.

[10] Pascalis O, Bachevalier J (1998). Face recognition in primates: a cross-species study. Behav Processes.

[11] Dufour V, Coleman M, Campbell R, Petit O, Pascalis O (2004). On the species-specificity of face recognition in human adults. Cahiers de Psychologie Cognitive/Current Psychology of Cognition.

[12] Rossion, B. & Michel, C. In *The Oxford Handbook of Face Perception* 215-244 (Oxford University Press, 2011).

[13] Malpass RS, Kravitz J (1969). Recognition for faces of own and other race. J Pers Soc Psychol.

[14] Carmel D, Bentin S (2002). Domain specificity versus expertise: Factors influencing distinct processing of faces. Cognition.

[15] de Haan M, Pascalis O, Johnson MH (2002). Specialization of neural mechanisms underlying face recognition in human infants. J Cognitive Neurosci.

[16] Itier RJ, Van Roon P, Alain C (2011). Species sensitivity of early face and eye processing. Neuroimage.

[17] de Haan M, Johnson MH, Halit H (2003). Development of face-sensitive event-related potentials during infancy: A review. Int J Psychophys.

[18] Hoehl S, Peykarjou S (2012). The early development of face processing — What makes faces special?. Neurosci Bull.

[19] Di Giorgio, E., Leo, I., Pascalis, O. & Simion, F. Is the face-perception system human-specific at birth? *Dev Psychol*, 10.1037/a0026521 (2011). 10.1037/a0026521 22142186

[20] Pascalis O, de Haan M, Nelson CA (2002). Is face processing species-specific during the first year of life?. Science.

[21] Scott, L. S. & Monesson, A. The origin of biases in face perception. *Psychol Sc*i **2**0, 676-680, doi:PSCI2348 [pii], 10.1111/j.1467-9280.2009.02348.x (2009). 10.1111/j.1467-9280.2009.02348.x 19422630

[22] Simpson EA, Varga K, Frick JE, Fragaszy D (2011). Infants experience perceptual narrowing for nonprimate faces. Infancy.

[23] Fair J, Flom R, Jones J, Martin J (2012). Perceptual learning: 12‐month‐olds’ discrimination of monkey faces. Child Dev.

[24] Peykarjou S, Pauen S, Hoehl S (2014). How do 9-month-old infants categorize human and ape faces? A rapid repetition ERP study. Psychophys.

[25] Halit H, de Haan M, Johnson MH (2003). Cortical specialisation for face processing: face-sensitive event-related potential components in 3- and 12-month-old infants. Neuroimage.

[26] Palermo R, Rhodes G (2007). Are you always on my mind? A review of how face perception and attention interact. Neuropsychologia.

[27] Fabre-Thorpe M, Delorme A, Marlot C, Thorpe S (2001). A limit to the speed of processing in ultra-rapid visual categorization of novel natural scenes. Journal of Cognitive Neuroscience.

[28] Thorpe S, Fize D, Marlot C (1996). Speed of processing in the human visual system. Nature.

[29] Rousselet GA, Macé MJM, Fabre-Thorpe M (2004). Animal and human faces in natural scenes: How specific to human faces is the N170 ERP component?. Journal of vision.

[30] Rossion B, Jacques C (2008). Does physical interstimulus variance account for early electrophysiological face sensitive responses in the human brain? Ten lessons on the N170. Neuroimage.

[31] Grill-Spector K, Kanwisher N (2005). Visual Recognition: As Soon as You Know It Is There, You Know What It Is. Psychol Sci.

[32] Rousselet GA, Mace MJ, Fabre-Thorpe M (2003). Is it an animal? Is it a human face? Fast processing in upright and inverted natural scenes. J Vis.

[33] Mack ML, Palmeri TJ (2015). The dynamics of categorization: Unraveling rapid categorization. J Exp Psychol Gen.

[34] Poncet M, Fabre-Thorpe M (2014). Stimulus duration and diversity do not reverse the advantage for superordinate-level representations: the animal is seen before the bird. Eur J Neurosci.

[35] Hershler O, Hochstein S (2005). At first sight: a high-level pop out effect for faces. Vision Res.

[36] Hershler O, Golan T, Bentin S, Hochstein S (2010). The wide window of face detection. J Vis.

[37] Crouzet SM, Kirchner H, Thorpe SJ (2010). Fast saccades toward faces: face detection in just 100 ms. J Vis.

[38] Reddy L, Wilken P, Koch C (2004). Face-gender discrimination is possible in the near-absence of attention. Journal of vision.

[39] Rossion, B., Torfs, K., Jacques, C. & Liu-Shuang, J. Fast periodic presentation of natural images reveals a robust face-selective electrophysiological response in the human brain. *Journal of vision* **15**, 10.1167/15.1.18 (2015). 10.1167/15.1.18 25597037

[40] Dzhelyova M, Rossion B (2014). The effect of parametric stimulus size variation on individual face discrimination indexed by fast periodic visual stimulation. BMC Neurosci.

[41] Liu-Shuang J, Norcia AM, Rossion B (2014). An objective index of individual face discrimination in the right occipito-temporal cortex by means of fast periodic oddball stimulation. Neuropsychologia.

[42] Langner O (2010). Presentation and validation of the Radboud FacesDatabase. Cog Emot.

[43] Tottenham, N. In *IL: John D. and Catherine T. MacArthur Foundation Research Network on Early Experience and Brain Development* (Chicago, 1998).

[44] Rossion, B. & Boremanse, A. Robust sensitivity to facial identity in the right human occipito-temporal cortex as revealed by steady-state visual-evoked potentials. *J Vis* **11**, 10.1167/11.2.16 (2011). 10.1167/11.2.16 21346000

[45] Rossion B (2014). Understanding face perception by means of human electrophysiology. Trends in Cognitive Sciences.

[46] Retter TL, Rossion B (2016). Uncovering the neural magnitude and spatio-temporal dynamics of natural image categorization in a fast visual stream. Neuropsychologia.

[47] Rouder JN, Morey RD, Speckman PL, Province JM (2012). Default Bayes factors for ANOVA designs. J Math Psychol.

[48] JASP (version 0. 8). https://jasp-stats.org/ (2016). [Computer Program]

[49] Jeffreys, H. *Theory of Probability*. 3rd edn, (Oxford University Press, 1961).

[50] Quinn PC, Eimas PD (1998). Evidence for a global categorical representation of humans by young infants. Journal of Experimental Child Psychology.

[51] Quinn PC (2004). Is the asymmetry in young infants’ categorization of humans versus nonhuman animals based on head, body, or global gestalt information?. Psychonomic Bulletin & Review.

[52] Marinovic V, Hoehl S, Pauen S (2014). Neural correlates of human-animal distinction: an ERP-study on early categorical differentiation with 4- and 7-month-old infants and adults. Neuropsychologia.

[53] Pauen S (2002). Evidence for knowledge-based category discrimination in infancy. Child Dev.

[54] Peykarjou, S., Wissner, J. & Pauen, S. Categorical erp repetition effects for human and furniture items in 7‐month‐old infants. *Infant Child Dev*, 10.1002/icd.2016 (2016).

[55] Behl-Chadha G (1996). Basic-level and superordinate-like categorical representations in early infancy. Cognition.

